# A metagenome-wide association study of gut microbiota in hepatitis B virus-related cirrhosis in northwest China

**DOI:** 10.3389/fgene.2025.1619911

**Published:** 2025-08-20

**Authors:** Meirong Feng, Yan Chai, Jinna Li, Qi Wang, Dekui Zhang

**Affiliations:** ^1^The Second Hospital and Clinical Medical School, Lanzhou University, Lanzhou, Gansu, China; ^2^Cuiying Biomedical Research Center, The Second Hospital and Clinical Medical School, Lanzhou University, Lanzhou, Gansu, China

**Keywords:** Hepatitis B virus-related cirrhosis, shotgun metagenomic sequencing, gut microbioal composition and function, metagenome-wide association study, Northwest China

## Abstract

**Background and purpose:**

In recent years, research on the relationship between hepatitis B virus-related cirrhosis (HBV-LC) and gut microbiota has grown, but studies focusing on the Northwest Chinese population are scarce. This study characterized the gut microbiota composition and function in HBV-LC patients vs. healthy individuals in Northwest China, aiming to provide a scientific basis for region-specific precision therapies.

**Materials and methods:**

A cross-sectional study enrolled 43 HBV-LC patients and 43 age-/sex-matched healthy controls (HC) from Gansu Province. Clinical parameters including liver function, blood routine, coagulation function, blood biochemistry were measured. Shotgun metagenomic sequencing was conducted to analyze gut microbiota taxonomic composition and function.

**Results:**

HBV-LC patients showed significantly elevated alanine aminotransferase (ALT), aspartate aminotransferase (AST), alkaline phosphatase (ALP), γ-glutamyl transferase (γ-GGT), prothrombin time, international normalized ratio (INR), and thrombin time, but reduced triglycerides (TG), total cholesterol (TC), erythrocytes, thrombocytes, total protein, albumin, and prothrombin time activity (PT-ratio). Alpha-diversity based on Shannon and Simpson indices was lower in HBV-LC. At the genus level, *Bacteroides*, *Prevotella*, *Escherichia*, *Parabacteroides*, *Veillonella*, and *Klebsiella* were enriched in HBV-LC, while *Bifidobacterium*, *Faecalibacterium, Roseburia*, *Ruminococcus*, *Anaerostipes*, *Blautia*, *Eubacterium*, and *Fusicatenibacter* were reduced. Species-level analysis identified distinct enrichment of *Prevotella copri, Bacteroides vulgatus, Escherichia coli, Fusobacterium nucleatum, and Veillonella* spp. in HBV-LC. Functional analysis revealed 482 metabolic pathways. HBV-LC showed enhanced lipid, amino acid, and nucleotide metabolism, menaquinol biosynthesis, and anaerobic energy metabolism, but reduced acetate/lactate production, lactose/galactose degradation, and peptidoglycan biosynthesis. Metagenome-wide association study revealed HBV-LC-enriched opportunistic species (e.g., *E. coli, Veillonella* spp.) correlated positively with hepatic enzymes and coagulation parameters, and negatively with TC, TG, and erythrocyte counts.

**Conclusion:**

HBV-LC patients in Northwest China exhibit altered clinical indicators, gut microbial composition (reduced diversity, increased opportunistic pathogens, decreased beneficial species), and metabolic function. These findings highlight the potential of gut microbiome-targeted interventions for regional precision medicine of HBV-LC.

## 1 Introduction

Hepatitis B virus (HBV) infection is a global public health concern, especially prevalent in Asia and Africa (Collaborators, 2022). As a high-endemic region for HBV, China has approximately 93 million chronic HBV carriers according to the Chinese Center for Disease Control and Prevention, with a significant proportion at risk of developing liver cirrhosis (LC), a chronic degenerative liver disease characterized by progressive hepatic fibrosis and functional decline, affects over 15 million individuals globally (Gines et al., 2021). HBV-related liver cirrhosis (HBV-LC), accounting for ∼60% of liver cirrhosis cases in China (Huang et al., 2023; Liu et al., 2024; Lou et al., 2024) (Huang et al., 2023; Liu et al., 2024; Lou et al., 2024), with immune-inflammatory mechanisms driving hepatocellular damage and fibrogenesis (Iannacone and Guidotti, 2022). HBV-LC severely compromises patients’ quality of life and survival prognosis, imposing a heavy burden on families and society (Sarin et al., 2016) In China, regional disparities in environmental factors, lifestyle habits, and genetic backgrounds may lead to variations in the pathogenesis and progression of HBV-LC (Ren et al., 2021).

Gansu Province, located in the northwest of China, features a unique geographical environment, dietary habits, and genetic background of its population. Although Gansu has a certain prevalence of HBV infection and liver cirrhosis, there is a paucity of metagenomic studies on the gut microbiota of HBV-LC patients in this region. The gut microbiota, referred to as the “second genome” of the human body, is intricately linked to overall health and disease (Fan and Pedersen, 2021). With the emerging concept of the gut-liver axis in recent years, the association between the gut microbiota and liver diseases has received extensive attention (Pabst et al., 2023). In HBV-related liver diseases, gut microbiota dysbiosis may influence disease development through multiple mechanisms. On one hand, portal hypertension in LC causes gastrointestinal congestion, reduced peristalsis, and impaired intestinal mucosal barrier function, increasing intestinal permeability. This allows bacteria and their metabolites, such as proinflammatory lipopolysaccharides (LPS), to translocate into the bloodstream and reach the liver via the portal vein, activating liver immune cells and triggering the release of cytokines including TNFα, IL-1, and IL-6. These cytokines disrupt the synthesis and degradation of extracellular matrix, thereby promoting liver fibrosis (Ohtani and Kawada, 2019; Hu et al., 2025). On the other hand, gut microbiota imbalance results in a decrease in beneficial bacteria, such as *Bifidobacterium* and *Lactobacillus*, while facilitating the overgrowth of potential pathogens, including *Veillonella* and Enterobacteriaceae, further exacerbating liver damage by disrupting the gut microecological balance (Milosevic et al., 2019).

The development of metagenomics, which enables direct sequencing of the genomes of all gut microorganisms without the need for bacterial culture, has provided a powerful tool for gut microbiota research. Compared to 16S rDNA sequencing strategies, shotgun metagenomic sequencing could comprehensively revealing the composition, structure, and predicting the function of the gut microbiota (Durazzi et al., 2021). In this study, we aim to comprehensively analyze the unique changes of the gut microbiota in HBV-related liver cirrhosis patients in Gansu through shotgun metagenomic sequencing and metagenome-wide association study (MWAS), clarify the relationship between gut microbiota and HBV-LC occurrence, progression, and prognosis, and lay a foundation for a deeper understanding of the pathogenesis of HBV-LC and the development of precision treatment strategies in Northwest China region.

## 2 Materials and methods

### 2.1 Subjects and study design

This study was conducted in compliance with the principles of the Declaration of Helsinki and approved by the Ethics Committee of Lanzhou University Second Hospital (Protocol Code: 2022A-824; Approval Date: 8 February 2022). 43 hospitalized patients with HBV-related cirrhosis (HBV-LC) and 43 age-/sex-matched healthy controls (HC) from Gansu Province were enrolled. Informed consent was obtained from all subjects before sample collection.

The inclusion criteria for HBV-LC patients were as follows: 1) Positive HBsAg persists for more than 6 months, exclude of acute HBV infection; 2) Imaging: CT showed nodules on the liver surface, widened liver fissures; 3) between 18–75 years old; 4) exclude other causes including alcohol intake (<20 g/day for men, <10 g/day for women), co-infections (HCV, HDV, and HIV), no autoimmune liver disease, drug-induced liver injury, or hereditary liver disease. 5) Child-Pugh: A, B, C grade to distinguish between compensation period and decompensation period. 6) No any other complications such as asces, esophageal and gastric fundus bleeding, or hepatic encephalopathy.

The inclusion criteria for the HC group: 1) matched age, sex, lifestyle with HBV-LC patients; 2) No evidence of HBV infection: HBsAg, anti-HBC and anti-HBs were all negative; 3) No liver diseases: Liver function indicators including ALT, AST, total bilirubin, albumin, and coagulation function are normal. 4) No metabolic diseases including diabetes, hypertension, hyperlipidemia, non-alcoholic fatty liver disease. 5) No gastrointestinal diseases, no history of major surgeries (especially abdominal surgeries).

Exclusion criterion for both groups: 1) malignant tumors (especially hepatocellular carcinoma), severe complications (Failure of heart, kidney and lung functions, infection such as Sepsis, tuberculosis), pregnant or lactating women; 2) Use of antibiotics, probiotics, laxatives and proton pump inhibitors in the past 4 weeks; 3) Use of immunomodulators in the past 3 months; 4) accepted intestinal surgery or endoscopic treatment in the past 1 month; 5) with chronic diarrhea or constipation.

Basic information, including age, sex, height, weight, BMI, residential address, medication history, bowel movement patterns, family history of liver disease, and lifestyle factors (e.g., smoking, alcohol consumption) were systematically collected in Supplementary Table S1 using a standardized questionnaire. Clinical indicators, including blood routine, coagulation function, liver function tests, blood biochemistry were collected from Lanzhou University Second Hospital (Supplementary Table S1). Fecal samples were collected from all the subjects using sterile containers, immediately snap-frozen at −80°C and were transported to the laboratory on dry ice for total DNA extraction and metagenomic sequencing after all the samples were collected.

### 2.2 DNA preparation and shotgun metagenomic sequencing

Total DNA was extracted from ice-thawed samples using the phenol/trichloromethane method. Total DNA extracts were treated with DNase-free RNase to remove RNA contamination, followed by quality assessment using agarose gel electrophoresis and quantification using a Qubit 3.0 fluorimeter (Thermo Fisher, Waltham, MA, United States). After total DNA passed quality control (concentration and integrity testing), 500 ng of DNA was fragmented into 300∼700 bp fragments by ultrasonic shearing with a Covaris E220 system (Covaris, Brighton, United Kingdom). Size selection of targeted fragments was performed using magnetic bead-based purification.

Subsequently, DNA fragments underwent end repair, adenylation of 3′ends, and ligation with indexed adapters. Ligated products were amplified by PCR, hybridized with exon-specific probes, and captured by streptavidin-coated magnetic beads. Captured DNA was subjected to a second round of PCR amplification, followed by circularization to generate single-stranded circular (ssCir) libraries. The ssCir libraries were amplified through rolling circle amplification (RCA) to obtain DNA nanoballs (DNBs), which were loaded onto a flowcell, and sequenced using the DNBSEQ T1 Platform (BGI-Shenzhen, China) using paired-end 150 bp reads (Durazzi et al., 2021; Fang et al., 2018).

An average of 7.0 Gb raw reads were obtained. The raw sequencing reads were firstly processed to filter low-quality reads (≥50% bases with Q ≤ 20) (Kultima et al., 2012) andadapter trimming using Fastp (Chen et al., 2018). Then the filtered reads were further used to remove human genome-derived reads using Bowtie2 (Langmead and Salzberg, 2012) against the hg38 reference genome. Finally an average of 6.90 Gb reads were retained and defined as high-quality microbial reads for further taxonomic and functional analysis.

### 2.3 Taxonomic and functional profiling acquisition

Taxonomic profiles were generated from high-quality final clean reads using Metaphlan 4.0 (Truong et al., 2015). The analysis was carried out with the following parameters: input_type fastq -ignore_viruses -nproc 6, as detailed on the official company website (MetaPhlAn4 - The Huttenhower Lab (harvard.edu)). This approach enabled the accurate identification and quantification of microbial taxa present in the samples.

For functional profiling, HUMAnN 4.0 was employed to comprehensively assess the abundance of microbial metabolic pathways and other molecular functions within the metagenomic sequencing data. The software was run with the parameters -i input_clean_data -o output--threads 10 --memory - use maximum--remove - temp - output, following the guidelines provided in the corresponding research paper (Beghini et al., 2021) and the official website (https://huttenhower.sph.harvard.edu/humann). This method allowed for an in - depth understanding of the functional capabilities of the microbial community.

### 2.4 Diversity calculation

Alpha diversity [within-sample diversity, R 4.0.3 vegan: diversity (data, index = “richness/shannon/Simpson/InSimpson”)] was calculated using the richness, Shannon index, Simpson index, and Inverse Simpson index, depending on the taxonomic profiles (Qin et al., 2012).

Beta diversity (between-sample diversity, R 4.0.3 ape: pcoa (“bray_curtis distance”, correction = “none”, rn = NULL), was calculated using the Bray-Curtis distances matrices derived from taxonomic profiles.

Permutational multivariate analysis of variance (PERMANOVA) was performed using the adonis () function in R (vegan v4.0.3), with the formula dist ∼ group and 9999 permutations, to assess the statistical significance of group differences in gut microbial composition.

### 2.5 Statistical analysis

All statistical analyses were performed using R statistical software (version 4.2.0; R Core Team, 2018) with Bioconductor 3.13, adjusting for covariates specified in prior sections (Huber et al., 2015). Clinical characteristics were compared between cirrhosis patients and healthy controls using Student’s t-test [t.test (data, group)] for continuous variables or Fisher’s exact test [fisher.test (data)] for categorical variables (e.g., gender).

Differential abundances of gut microbes (at species, genus, and phylum levels) were identified using the Wilcoxon rank-sum test, with significant results further validated by Storey’s false discovery rate (FDR) correction for multiple comparisons. The association between microbial taxa and host metabolites was evaluated via Spearman’s rank correlation coefficient. P value <0.05 was considered statistically significant.

Principal coordinate analysis (PCoA) of microbial beta diversity was conducted using the pcoa function in the ape package (R version 4.3.1). For metabolomics data, differentially regulated metabolites between groups were identified via Wilcoxon rank-sum test (P < 0.05), followed by Benjamini–Hochberg correction for multiple testing using the p.adjust (p.value, method = “BH”) function.

## 3 Results

### 3.1 Phenotypic and clinical indicators analysis of all the subjects

A total of 86 participants were enrolled and categorized into a healthy control (HC) group and a hepatitis B virus-related liver cirrhosis (HBV-LC) group and their clinical indicators were analyzed in Table 1. The male-female ratio (Fisher’s exact test), age and BMI (Student’s t-test and wilcoxon rank-sum test) between two groups showed no significant differences (P > 0.05, Supplementary Figure S1; Table 1). Compared with HC subjects, HBV-LC patients exhibited significantly lower levels of erythrocyte and thrombocyte (P < 0.05). Coagulation function tests revealed prolonged prothrombin time, international normalized ratio (INR), and thrombin time, accompanied by reduced PT-ratio in the HBV-LC group (P < 0.05). Liver function markers showed significantly elevated alanine aminotransferase (ALT), aspartate aminotransferase (AST), alkaline phosphatase (ALP), and γ-glutamyl transferase (γ-GGT) in HBV-LC patients versus HC controls (P < 0.05). Conversely, total protein, albumin, triglycerides (TG), and total cholesterol (TC) were significantly decreased in the HBV-LC group (P < 0.05). No significant group differences were observed for activated partial thromboplastin time (APTT), D-dimer, or fibrinogen degradation products (FDP) (P > 0.05 for all; Table 1; Supplementary Figure S2).

**TABLE 1:** The demographical and clinical indicators of patients with HBV-LC patients and healthy subjects.

Parameters	HBV-LC group	HC group	Statistical test			
			Student’s t-test		Wilcoxon rank-sum test	
			P Value	Q value	P Value	Q value
Gender (M:F)	16:27	17:26	1 (fisher.test)	1.00		
Age	50.40 ± 9.69	51.19 ± 8.58	0.690	0.704	0.846	0.846
BMI	22.17 ± 1.79	22.44 ± 1.55	0.450	0.503	0.223	0.265
Erythrocyte	4.43 ± 0.81	4.98 ± 0.72	1.32E-03	2.49E-03	6.22E-04	1.18E-03
Thrombocyte	96.23 ± 61.04	216.64 ± 63.63	7.42E-14	7.05E-13	6.40E-11	6.08E-10
Total protein	68.07 ± 7.67	72.72 ± 5.06	1.44E-03	2.49E-03	5.63E-03	7.64E-03
Albumin	38.96 ± 5.87	43.21 ± 4.40	2.91E-04	6.90E-04	4.60E-04	9.72E-04
ALT	68.86 ± 123.01	21.12 ± 12.79	1.51E-02	2.21E-02	3.26E-06	1.03E-05
AST	82.72 ± 161.45	25.44 ± 7.95	2.50E-02	3.40E-02	1.23E-06	5.86E-06
ALP	120.21 ± 72.13	85.35 ± 17.37	3.45E-03	5.46E-03	2.19E-03	3.47E-03
γ-GGT	75.77 ± 87.55	21.28 ± 12.15	2.12E-04	5.74E-04	2.44E-06	9.26E-06
Prothrombin time	13.78 ± 2.96	11.31 ± 0.82	3.16E-06	2.00E-05	6.48E-09	4.10E-08
PT-ratio	78.78 ± 23.92	95.53 ± 13.16	1.51E-04	5.00E-04	1.14E-03	1.97E-03
INR	1.18 ± 0.26	1.04 ± 0.08	1.44E-03	2.49E-03	2.38E-03	3.47E-03
APTT	32.28 ± 5.99	32.67 ± 2.74	0.704	0.704	0.300	0.335
Thrombin time	18.84 ± 2.22	14.09 ± 0.79	2.44E-18	4.64E-17	1.42E-15	2.70E-14
D-dimmer	1.10 ± 1.75	0.48 ± 1.16	0.0532	0.0673	0.182	0.230
FDP	3.06 ± 4.01	2.11 ± 4.81	0.324	0.385	0.720	0.760
TG	0.90 ± 0.50	1.67 ± 1.08	1.58E-04	5.00E-04	3.11E-05	8.43E-05
TC	3.43 ± 0.96	4.51 ± 0.81	9.16E-06	4.35E-05	3.72E-05	8.83E-05

Note: ALT: alanine aminotransferase; AST: aspartate aminotransferase; ALP: alkaline phosphatase; γ-GGT: γ–glutamyltransferase; PT-ratio: prothrombin time activity; INR: international normalized ratio; APTT: activated partial prothrombin time; FDP: fibrinogen degradation products; TG: triglycerides; TC: Total cholesterol. All the clincial indicators were calcuated as mean ± SD., Gender was analyzed with Fisher’s exact test, all the other clinical indicators were analyzed by Student’s t-test and wilcoxon rank-sum test.

### 3.2 Gut microbiota composition and diversity analysis

**FIGURE 1:**
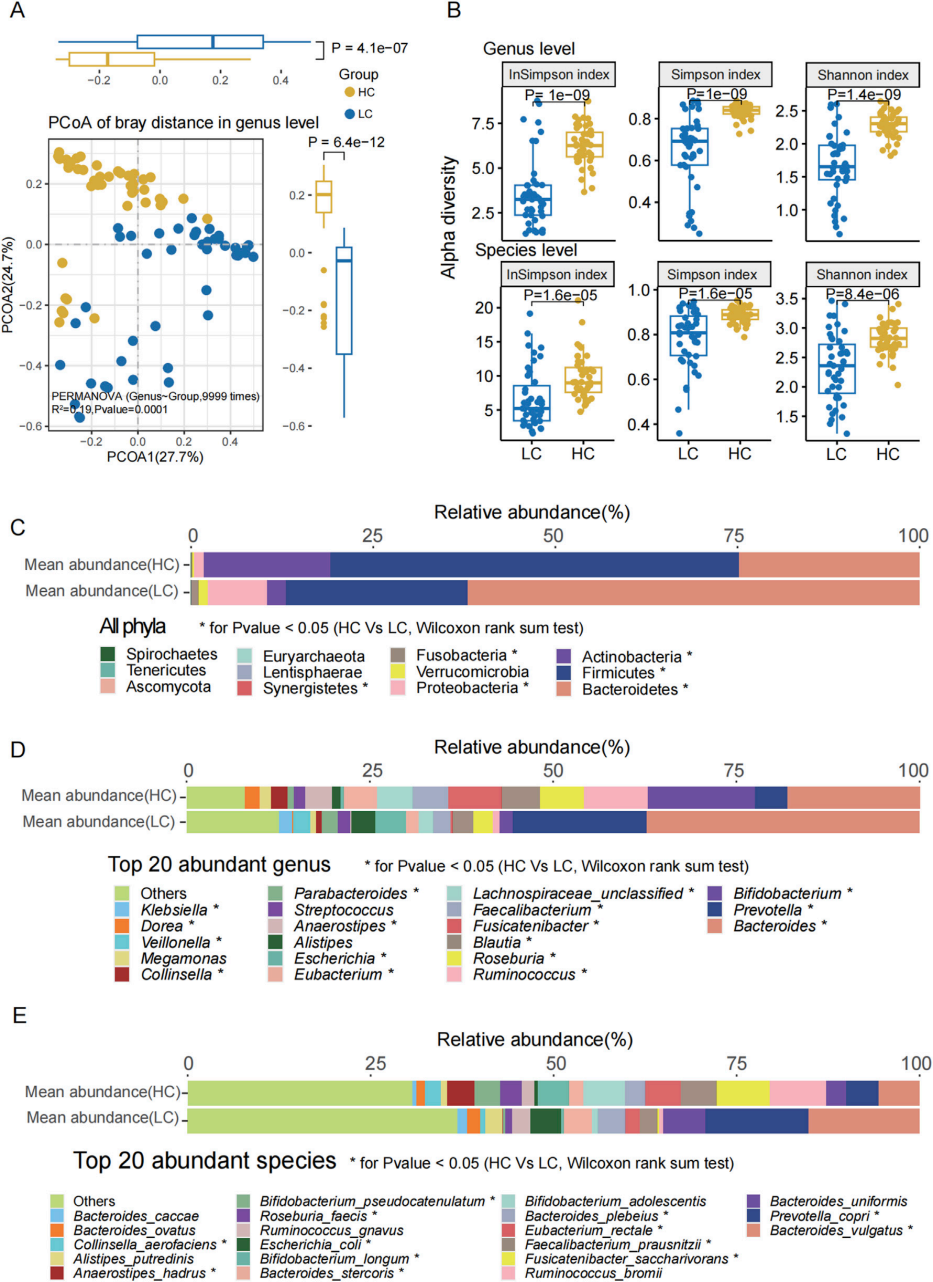
Gut microbiota composition and diversity differences between groups. **(A)** PCoA based on Bray-Curtis distance at the genus level revealed significant differences between the two groups (P < 0.0001, PERMANOVA). **(B)** Alpha diversity indices (Shannon, Simpson, and Inverse Simpson) revealed significantly reduced microbial richness and diversity in the HBV-LC (LC) group compared to HC at both genus and species levels (P < 0.001 for all). **(C)** Relative abundance distribution of all the bacterial phyla in both groups. **(D)** The top 20 abundant genera ranked by mean relative abundance. **(E)** The top 20 abundant species ranked by mean relative abundance. Statistical significance: * denotes P < 0.05, ** denotes P < 0.01, *** denotes P < 0.001 (Wilcoxon rank-sum test, FDR correction).

To investigate the differences in gut microbial communities, we conducted shotgun metagenomic sequencing on the samples and performed taxonomic annotation. MetaPhlan 4.0 was used to annotate 12 phyla, 182 genera, and 590 species for all the samples (Supplementary Table S3). Significant disparities in the gut microbiome were detected between the HBV-LC and HC groups using PERMANOVA (Bray-curtis distance at genus and species) and Wilcoxon rank-sum test (Figure 1).

At the genus level, PCoA based on the Bray-Curtis distances indicated a significant difference between the two groups (Figure 1A, *P* = 0.0001, R^2^ = 0.19, PERMANOVA; Supplementary Table S4). The Shannon (P = 1e^−9^), Simpson (P = 1e^−9^), and Inverse Simpson (P = 1.4e^−9^) indices of the HBV-LC group were significantly lower than those of the HC group (Figure 1B). Similarly, at the species level, PCoA analysis based on the Bray-Curtis distances also showed a significant difference between the two groups (P = 0.0001, Supplementary Figure S3). The Shannon (P = 1.6e^−5^), Simpson (P = 1.6e^−5^), and Inverse Simpson (P = 8.4e^−6^) indices of the HBV-LC patients were significantly lower compared to the HC group (Figure 1B; Supplementary Table S5).

Subsequently, we analyzed all the phyla, the top 20 most abundant genera, and species (Figures 1C–E). Among the most abundant phyla, the relative abundances of Bacteroidetes, Proteobacteria, Fusobacteria, and Synergistetes were significantly higher in the HBV-LC group, whereas those of Firmicutes and Actinobacteria were significantly higher in the HC group.

Among the top 20 most abundant genera, the relative abundance of *Bacteroides*, *Prevotella*, *Escherichia*, *Parabacteroides*, *Veillonella*, *Klebsiella* were significantly higher (P < 0.05) in the HBV-LC group. Conversely, *Bifidobacterium*, *Ruminococcus, Roseburia*, *Blautia*, *Fusicatenibacter*, *Faecalibacterium*, *Lachnospiraceae_unclassified*, *Eubacterium*, *Anaerostipes*, *Collinsella*, *Dorea* were significantly enriched in the HC group.

For the top 20 most abundant species, *Veillonella dispar, Veillonella atypica, Veillonella* sp. T11011_6*, Prevotella copri, Veillonella parvula, Fusobacterium nucleatum, Haemophilus parainfluenzae, Campylobacter concisus, Escherichia coli* and *Aggregatibacter segnis* were significantly enriched in HBV-LC group. Compared with HBV-LC group, the *Bifidobacterium longum, Fusicatenibacter saccharivorans, Eubacterium hallii, Anaerostipes hadrus, Blautia wexlerae, Dorea longicatena, Blautia obeum, Dorea formicigenerans, Intestinibacter bartlettii, Eubacterium ramulus*, and *Coprococcus catus* were significantly enriched in HC group.

**FIGURE 2:**
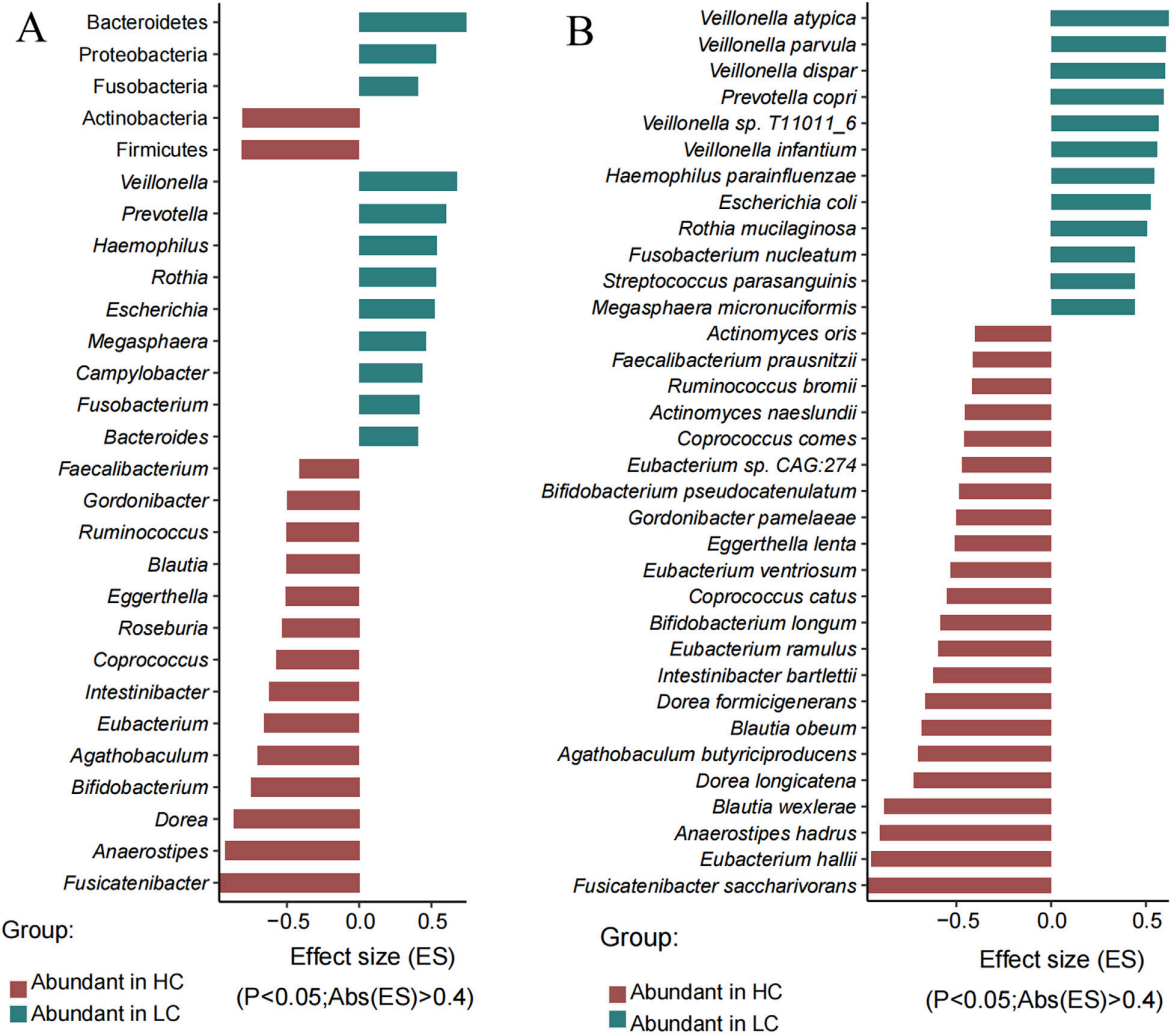
Significantly different phyla, genera and species between the HC and HBV-LC (LC) groups determined by Wilcoxon rank-sum test (P < 0.05, |effect size| > 0.4). **(A)** Significantly different phyla (top five no italic) and genera (italic). **(B)** Significantly different species. Red indicates taxa with higher abundance in the HC group, while green denotes taxa more abundant in the HBV-LC group. Note: only the absolute effect size over 0.4 were showed here due to space, the whole differential phyla, genera and species were showed in Supplementary Table S3.

To further reveal the differences of the gut microbes between two groups, we analyzed all the significantly different genera and species between HBV-LC and HC groups using wilcoxon rank-sum test. Totally six differential phyla, 62 differential genera (33 genera were higher in HBV-LC group), and 148 differential species (88 species were higher in HBV-LC group) were observed between two groups (P < 0.05, Supplementary Table S3). Figure 2 showed the significantly different phyla, genera, and species according to the effective size of each taxa (P < 0.05, |effective size|>0.4). At the genus level, except for the high abundant *Bacteroides*, *Prevotella*, *Escherichia*, *Parabacteroides*, *Veillonella* and *Klebsiella*, *Fusobacterium, Campylobacter, Megasphaera, Rothia, Haemophilus* were also significantly enriched in the HBV-LC group, while *Fusicatenibacter, Anaerostipes, Dorea, Bifidobacterium, Agathobaculum, Eubacterium, Intestinibacter, Coprococcus, Roseburia, Eggerthella, Blautia, Ruminococcus, Gordonibacter, Faecalibacterium* were significantly enriched in the HC group (Figure 2A).

At the species level, *Veillonella dispar* (P = 3.05E-07),*Prevotella copri* (P = 1.12E-06), *Fusobacterium nucleatum* (P = 1.56E-06), *Megasphaera micronuciformis* (P = 8.38E-06), *Campylobacter concisus* (P = 1.53E-05), *Haemophilus parainfluenzae* (P = 1.13E-05), *Campylobacter gracilis* (P = 1.53E-05), *Escherichia coli* (P = 3.24E-05), *Rothia mucilaginosa* (P = 3.72E-05), and *Aggregatibacter segnis* (P = 5.09E-05) were significantly abundant in the HBV-LC group, while *Fusicatenibacter saccharivorans* (*P* = 1.36E-14), *Eubacterium hallii* (P = 3.63E-14), *Aaerostipe hadrus* (P = 6.49E-13), *Agathobaculum butyriciproducens* (P = 2.43E-08), *Intestinibacter bartlettii* (P = 5.49E-07), *Eubacterium ramulus* (P = 1.57E-06), *Actinomyces naeslundii* (P = 2.11E-06), *Bifidobacterium longum* (P = 3.55E-06), *Coprococcus catus* (P = 9.97E-06) and *Eubacterium ventriosum* (P = 1.99E-05) were significantly enriched in the HC group (Figure 2B; Supplementary Table S3).

Notably, we observed an abnormal increase in *Lactobacillus* abundance among HBV-related liver cirrhosis (HBV-LC) patients compared with healthy subjects (Supplementary Table S8). Further species-level analysis revealed that three specific species—*Lactobacillus mindensis, Lactobacillus salivarius, and Lactobacillus sanfranciscensis*—were significantly enriched in the HBV-LC group (P < 0.05).

### 3.3 Metagenomic functional pathway analysis

**FIGURE 3:**
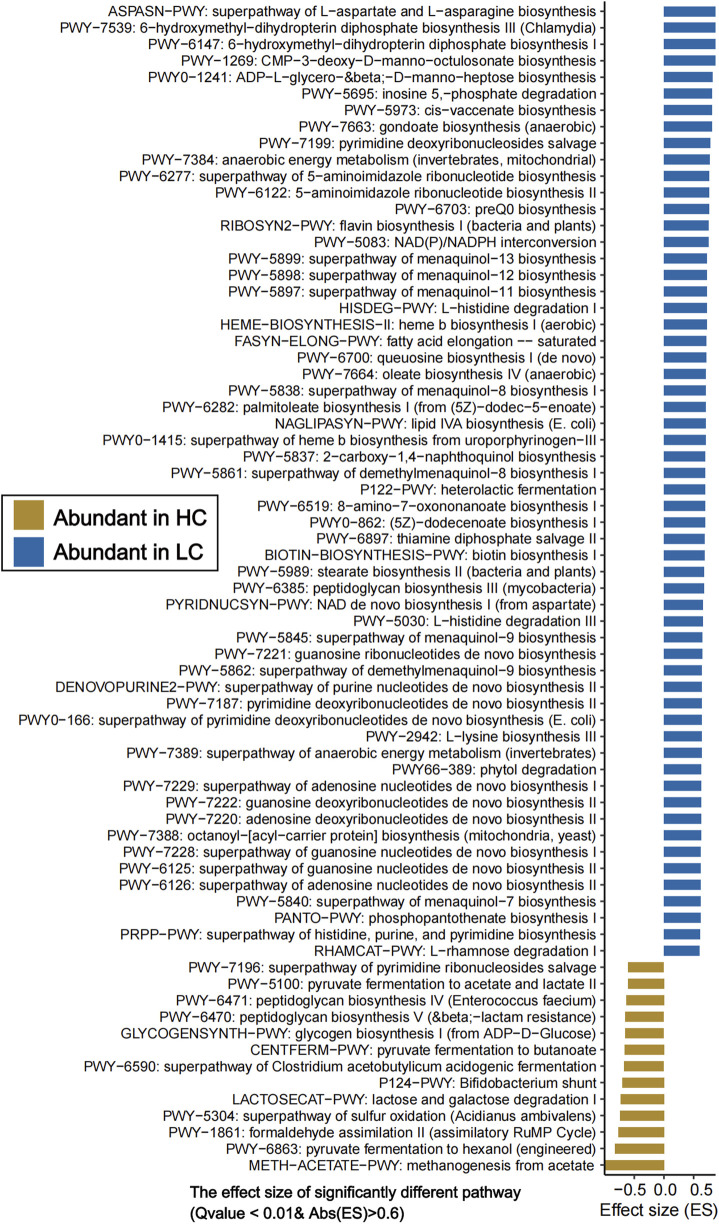
Significantly different gut microbiota functional pathways between HC and HBV-LC (LC) groups (Q value <0.01, |effect size| > 0.6). The horizontal axis denotes “effect size” of each functional pathway, and the vertical axis denotes “functional pathways”. Pathways enriched in the HC group are highlighted in yellowish-brown, whereas those enriched in HBV-LC patients are marked in blue.

To further investigate the gut microbial function, HUMAnN 4.0 was used to efficiently and accurately profile the abundance of microbial metabolic pathways and other molecular functions from shotgun metagenomic sequencing data (Supplementary Table S6). PCoA showed significant differences in the gut microbiota functional pathways between HBV-LC patients and HC subjects (P = 0.001, PERMANOVA, Supplementary Figure S4). A total of 482 uniref pathways, and 22,032 gut microbiota metabolic pathways networks were annotated. Among the 482 pathways, 250 pathways were significantly enriched in HBV-LC group, while 50 pathways showed significant enrichment in HC groups (P < 0.05, Supplementary Table S6). Figure 3 showed the significantly different functional pathways (Q value <0.01 & |effect size|> 0.6). The HBV-LC group were mainly enriched in lipid metbaolism (e.g., lipid IVA biosynthesis, oleate biosynthesis, saturated fatty acid elongation, stearate biosynthesis II, palmitoleate biosynthesis, fatty acid beta-oxidation), amino acids biosynthesis (L-aspartic acid, L-asparagine, and L-lysine biosynthesis, L-histidine degradation, branched chain amino acid biosynthesis), *de novo* nucleotides biosynthesis (superpathway of adenosine/guanosine/purine nucleotides *de novo* biosynthesis, guanosine/adenosine/pyrimidine deoxyribonucleotides *de novo* biosynthesis, 5-aminoimidazole ribonucleotide biosynthesis), menaquinol biosynthesis, and anaerobic energy metabolism pathways. Conversely, the HC group exhibited enrichment of pathways including methanogenesis from acetate, pyruvate fermentation to hexanol (engineered), lactose/galactose degradation I, formaldehyde assimilation II (assimilatory RuMP cycle), glycogen biosynthesis I (from ADP-D-glucose), sulfur oxidation superpathway (Acidianus ambivalens), *Bifidobacterium* shunt, peptidoglycan biosynthesis, pyruvate fermentation to acetate and lactate. Notably, both groups showed high abundance of functional pathways associated with nutrient metabolism (e.g., carbohydrate metabolism), though the specific enriched pathways differed significantly between HBV-LC and HC group (Figure 3; Supplementary Table S3).

### 3.4 Metagenome-wide association study

**FIGURE 4:**
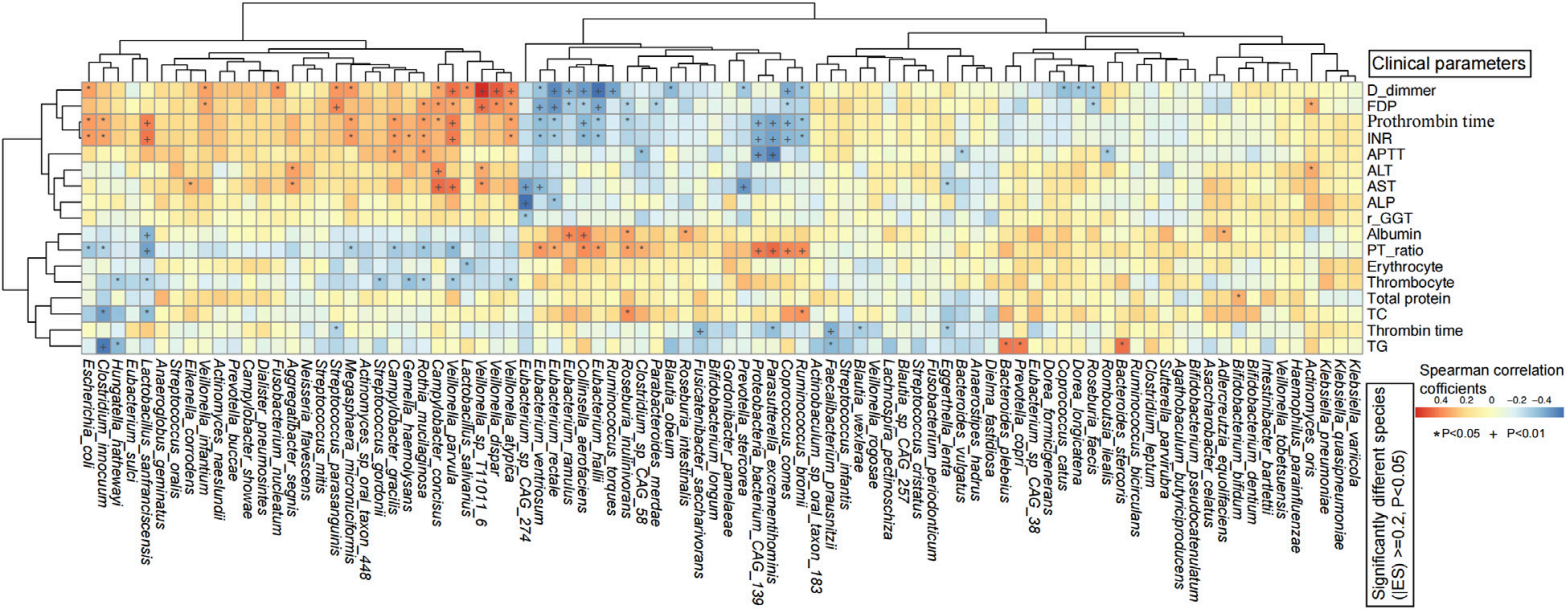
Spearman’s rank correlation analysis between significantly differential species and clinical indices of all subjects revealed significant associations between gut microbes and clinical parameters. The horizontal axis represents the significant differential gut bacteria, and the vertical axis represents the clinical indicators. The color bar column depicts the spearman’s correlation coefficients. *P < 0.05, + P < 0.01.

To further elucidate the relationship between the clinical indicators related to HBV-related liver cirrhosis and gut microbiota, metagenome-wide association study was performed for all the 86 samples. Significant correlations between certain clinical parameters and specific microbial species were observed (Figure 4; Supplementary Table S7).

In the HBV-LC group, coagulation indicators including D-dimmer, FDP, prothrombin time, INR, and APTT were significantly positively associated with HBV-LC-related opportunistic species including *Fusobacterium nucleatum*, *Streptococcus parasanguinis*, *Campylobacter concisus*, *Veillonella infantium*, *Veillonella parvula*, *Veillonella dispar*, *Veillonella atypica*, but were negatively correlated with HBV-LC-reduced species including *Eubacterium ventriosum*, *E. rectale, E, ramulus*, *E. hallii*, *Collinsella aerofaciens*, *Roseburia faecis*, *Ruminococcus bromii*, *Parasutterella excrementihominis*, and *Coprococcus comes*. Thrombin time was significantly negatively associated with *Eggerthella lenta, Blautia wexlerae, Faecalibacterium prausnitzii, P. excrementihominis,* and *Fusicatenibacter saccharivorans*. PT-ratio was significantly positively associated with the above HBV-LC decreased species including *P. excrementihominis*. AST and ALT were significantly positively associated with *Aggregatibacter segnis* and *C. concisus*. Erythrocyte and Thrombocyte were negatively associated with *Streptococcus gordonii, Gemella haemolysans, Rothia mucilaginosa*, and *V. parvula*. TG was positively correlated with *Bacteroides plebeius, Bacteroides stercoris,* and *Prevotella copri*, but significantly negatively associated with *Clostridium innocuum* and *Hungatella hathewayi*. TC was significantly positively associated with *R. bromii* and *Roseburia inulinivorans*, but was negatively associated with *C. innocuum* and *L. sanfranciscensis*. Albumin was significantly positively associated with *Eubacterium ramulus, C. aerofaciens, R. inulinivorans, Roseburia intestinalis,* but was negatively associated with *L. sanfranciscensis*. Thrombin time was significantly negatively associated with *F. prausnitzii* (Figure 4).

In addition, for correlation with all 86 samples, high concentrations of ALT, AST, γ-GTT, and D-dimmer, as well as thrombin time, prothrombin time, international normalized ratio were significantly positively correlated with HBV-LC enriched species including *P. copri*, *P. buccae*, *Veillonella* spp. (e.g., *V. parvula*, *V. dispar*, *V.atypica*, *V. infantium*, *Veillonella rogosae*, *Veillonella tobetsuensis*), *Streptococcus* spp. (e.g., *S._cristatus*, *Streptococcus infantis*, *Streptococcus mitis*, *S. gordonii*, *S. parasanguinis*, *Streptococcus oralis*), *Escherichia coli*, *C. concisus*, *C. gracilis*, *R. mucilaginosa*, *A. segnis*, *Megasphaera micronuciformis*, *F. nucleatum, F. periodonticum*, *Neisseria flavescens*, *Anaeroglobus geminatus*, *Dialister pneumosintes*, *Campylobacter showae, Haemophilus parainfluenzae*, *G. haemolysans*, *Eikenella corrodens* etc., but were significantly negatively associated with the well-known beneficial species including *Bifidobacterium longum*, *B. pseudocatenulatum*, *B. bifidum*, *F. prausnitzii*, *Ruminococcus bicirculans*, *Eubacterium rectale* etc. (P < 0.05). These results suggested that there are significant positive correlations between high levels of clinical indicators in HBV-related liver cirrhosis patients and HBV-LC-enriched microbial species, the majority of which are pathogenic bacteria. Conversely, high levels of TC, TG, thrombocyte, erythrocyte, albumin, and prothrombin time activity showed significant positive correlations with HC-abundant gut microbial species including Fusicatenibacter saccharivorans, *Eubacterium* spp. (e.g., *E. hallii, E. ventriosum, E. ramulus, E. rectale*), *Dorea longicatena*, *D. formicigenerans*, *Coprococcus catus*, C. comes, *Gordonibacter pamelaeae* etc., whereas significant negative associations with HBV-LC-enriched *V. atypica*, *Campylobacter gracilis*, *E. coli*, *F. nucleatum*, *M. micronuciformis* etc. (Supplementary Figure S5)

Specially, for the two *Lactobacillus* spp. highly enriched in the HBV-LC group, *L. sanfranciscensis* was significantly positively correlated with thrombin time, prothrombin time, AST and INR, but was negatively correlated with thrombocyte, prothrombin time activity, albumin and TC. *Lactobacillus salivarius* was significantly positively associated with prothrombin time, γ-glutamyl transferase, and D-dimmer, but was significantly negatively associated with thrombocyte, prothrombin time activity, and erythrocyte.

These findings suggested significant associations between gut microbiota composition and clinical biochemical markers in HBV-related liver cirrhosis patients, reminding us that changes of the gut microbial composition could predicting HBV-related liver cirrhosis in terms of clinical indicators such as thrombocyte, thrombin time, albumin, prothrombin time, γ-glutamyl transferase, AST, ALT, TC and TG.

## 4 Discussion

This study systematically characterized the composition and function of the gut microbiota in patients with hepatitis B virus-related liver cirrhosis (HBV-LC) from Gansu Province, Northwest China using shotgun metagenomic sequencing for the first time, revealing significant alterations in clinical indicators, gut microbial composition, gut microbial metabolic pathways, as well as the relationship between gut microbiota and clinical parameters, which provide important evidence for regional precision diagnosis and treatment of HBV-related liver cirrhosis.

Clinical indicators including liver function, blood routine, coagulation function, and blood biochemistry showed varying degrees of abnormalities, indicating the lliver function disorders in HBV-LC patients. HBV-LC patients exhibited typical hematological features of cirrhosis, including significantly reduced erythrocyte, and thrombocyte, which were consistent with hypersplenism and anemia caused by portal hypertension in liver cirrhosis (Kobayashi et al., 2001; Amitrano et al., 2002). In addition, HBV-LC patients are accompanied with material and energy metabolism disorders. Liver function indicators, including ALT, AST, ALP, and γ-GGT were significantly elevated in patients with HBV-LC, reflecting hepatocellular injury and cholestasis, which might be correlated with persistent viral replication and progressive liver fibrosis (Shoaib et al., 2023; Vieira da Rocha et al., 2009). Notably, reduced erythrocyte, total protein, albumin, TC and TG were observed in the HBV-LC group, which were closely associated with impaired hepatic synthetic function (Kobayashi et al., 2001), nutritional malabsorption, and ascites formation (Wang et al., 2017; Ramcharran et al., 2011). In addition, coagulation abnormalities such as prolonged prothrombin time (PT), international normalized ratio (INR), and thrombin time (TT) were also significantly elevated in HBV-LC patients, which might attribute to insufficient hepatic synthesis of coagulation factors (Aiza-Haddad et al., 2024; Lisman et al., 2021)

The gut microbiota were reported to influence the progression of hepatitis B cirrhosis. In a Southeastern China study, Ren et al. reported fecal microbiota transplantation could induce hepatitis B virus e-antigen (HBeAg) clearance in patients with positive HBeAg after long-term antiviral therapy (Ren et al., 2017), verifying the important role of gut microbiota in HBV-LC, however, similar studies were lack in Northwestern China. In our study, we found significantly reduced gut microbial α diversity, increased Bacteroidetes, Proteobacteria, Fusobacteria, Synergistetes, *Bacteroides*, *Alistipes, Escherichia, Parabacteroides, Klebsiella*, and potential pathogenic bacteria such as *E. coli* and *Klebsiella pneumoniae*, decreased phyla Firmicutes and Actinobacteria, lower Firmicutes/Bacteroidetes ratio, and higher Proteobacteria in HBV-LC patients, aligns with known dysbiosis contributing to endotoxemia and were consistent with previously 16S rDNA sequencing results related to HBV-LC (Ren et al., 2017; Shi et al., 2025; Shu et al., 2022; Wang et al., 2024). In a recent study, Wang et al. reported that probiotics (*B. longum*, *Lactobacillus acidophilus*, and *E. faecalis*, BLE) could significantly promote the decline of hepatitis B surface antigen (HBsAg) and inhibit HBV replication by enhancing intestinal homeostasis and provoking intrahepatic interferon (IFN)-g+CD4^+^ T cell immune response. We have also observed a significant decrease of beneficail species such as *B. adolescentis*, *B. longum*, *B. pseudocatenulatum*, and *Eubacterium rectale.*

However, genus *Lactobacillus* and three of its species (totally 28 *Lactobacillus* spp. were detected) including *Lactobacillus mindensis*, *Lactobacillus salivarius*, and *L. sanfranciscensis* were significantly increased in the HBV-LC patients in our study. This was consistent with a previous study, in which *Lactobacillus* was higher in decompensated cirrhosis group compared with healthy and compensated groups (Shu et al., 2022). *L. mindensis* and *L. sanfranciscensis* are the predominant species in sourdoughs (Ehrmann et al., 2003; Lhomme et al., 2015). *L. salivarius* is a probiotic species with oropharyngeal tract resistance and adhesion to the oral epithelial cells (Yang et al., 2022), can stimulate immune cells to secrete anti-allergic related cytokines and regulate gastrointestinal function. We hypothesize that the increased abundance of *Lactobacillus* and its species in HBV-LC patients may be attributed to either specific dietary habits (e.g., people in Lanzhou take fermented steamed buns as main food) or a self-regulating protective mechanism evolved by the gut microbiota to inhibit pathogenic bacteria (Shu et al., 2022; Alexandrescu et al., 2024). Meanwhile, according to the correlation analysis between these *Lactobacillus* spp. and clinical indicators, the abnormal increase of these species in HBV-LC patients might due to the high levels of thrombin time, prothrombin time, AST, γ-glutamyl transferase, and international normalized ratio, but reduced levels of thrombocyte, TC, albumin, erythrocyte, and prothrombin time activity.

Notably, the relative abundance of SCFA-producing beneficial bacteria such as *E. rectale, E. hallii, Faecalibacterium prausnitzii, Bifidobacterium adolescentis*, *B. longum*, *B. pseudocatenulatum*, *Anaerostipes hadrus*, *Roseburia faecis, Ruminococcus bromii, Dorea longicatena, Blautia obeum, Blautia wexlerae*, *Coprococcus catus, Coprococcus comes, Gordonibacter pamelaeae, Agathobaculum butyriciproducens* were significantly decreased in HBV-LC patients. Meanwhile, *Fusicatenibacter saccharivorans,* a sugar fermenter to produce lactic acid, formic acid, acetic acid and succinic acid (Takad et al., 2013), was also significantly reduced in HBV-LC patients in our study, these results were consistent with a previous Japan study, in which decreased level of SCFAs is positively correlated with the degree of HBV-LC, and supplementation of butyrate can inhibit HBV replication (Honda et al., 2025).

Interestingly, 41 *Prevotella* spp. were detected, in which 22 were significantly increased in HBV-LC patients in our study, however was decreased in another HBV-LC related gut microbial study from Xiamen, Sountern China (Chen et al., 2020). *P. copri* was significantly positively associated with higher levels of thrombin time, prothrombin time, γ-glutamyl transferase, AST and ALT in HBV-LC patients, but negatively associated with HC-enriched thrombocyte. *P. stercorea* showed significantly negative associations with total protein. *P. buccae* showed significantly positive correlations with thrombin time, prothrombin time,γ-glutamyl transferase, AST, international normalized ratio, D-dimmer, but significantly negative associations with thrombocyte, TC, TG, and Prothrombin time activity. These results reminded us that the changes in gut microbiota in HBV-LC patients might be due to the alterations of haematological indexes, but evidence is needed to prove the causal relationship between clinical indicators and the gut microbiota.

Additionally, *Veillonella* and its species including *V. atypica, V. dispar, V infantium, V. parvula, Veillonella rodentium, Veillonella rogosae, V.* sp. T11011_6*, Veillonella tobetsuensis* were significantly increased in HBV-LC patients in our study, but were not reported in aother HBV-LC study (Shu et al., 2022). *Veillonella* is part of the normal flora of the mouth, gastrointestinal tract and vagina. *Veillonella* spp. are often regarded as pathogens and are associated with oral infections; bite wounds; head, neck, and various soft tissue infections; serious gonococcal infection; septic arthritis and meningitis; and infections of the sinuses, lungs, heart, bone, and CNS (Rojas-Tapias et al., 2022; Brook, 1996; Gouze et al., 2019; Rovery et al., 2005). These results suggested that increased relative abundance of gut microbial *Veillonella* spp. might be biomarkers of HBV-LC in Northwest China, where people eat high-salt, pasta/pickled foods, but further molecular studies are needed to verify the conclusion.

Functional metabolic analysis showed that the HBV-LC group’s microbiota was significantly enriched in lipid metabolism, amino acids biosynthesis, de novonucleotides biosynthesis, menaquinol biosynthesis, and anaerobic energy metabolism pathways, which may represent a feedback regulatory mechanism in response to the disrupted hepatic substance and energy metabolism in HBV-LC patients. As the liver serves as the primary organ responsible for material and energy metabolism, LC patients typically exhibit disturbances in the metabolism of the three major nutrients (carbohydrates, lipids, and proteins) as well as energy metabolism. Specifically, clinical manifestations include impaired glucose tolerance, hepatogenic diabetes, and an increased risk of hypoglycemia; disorders in fatty acid oxidation; altered amino acid profiles characterized by elevated plasma levels of aromatic amino acids and decreased levels of branched-chain amino acids; and abnormalities in energy metabolism such as elevated basal metabolic rate, depletion of energy reserves, and impaired energy utilization.

For lipid metabolism, the HBV-LC group’s microbiota mainly participated in lipid IVA biosynthesis, oleate biosynthesis, saturated fatty acid elongation, stearate biosynthesis II, palmitoleate biosynthesis, fatty acid beta-oxidation, fatty acid elongation, superpathway of fatty acid biosynthesis initiation, which exacerbated intrahepatic inflammation and changed fatty acid metabolism, forming a metabolic negative feedback with decreased clinical lipid levels, reminding us that microbial metabolic abnormalities may contribute to cirrhosis-related lipid disorders (Peng et al., 2023; Rudler et al., 2021). For amino acid metabolism, elevated L-aspartic acid, L-asparagine, and L-lysine biosynthesis, and L-histidine degradation, increased superpathway of branched chain amino acid biosynthesis and decreased superpathway of aromatic amino acid biosynthesis were observed in the HBV-LC group, which may be linked to enhanced metabolic activity of specific bacterial species, however, whether the changes of these amino acid metabolism were related to ammonia detoxification requires further verification (Rudler et al., 2021; Chen et al., 2014). For the nucleotides metabolism, enhanced nucleotide catabolism including superpathway of adenosine/guanosine/purine nucleotides *de novo* biosynthesis, guanosine/adenosine/pyrimidine deoxyribonucleotides *de novo* biosynthesis, 5-aminoimidazole ribonucleotide biosynthesis was enriched in the HBV-LC group released ammonia and inflammatory signals, potentially synergizing with the progression of hepatic encephalopathy and providing new evidence for the microbiota-brain axis mechanism (Rudler et al., 2021). Additionally, the superpathway of menaquinol (vitamin K2) was found to be significantly upregulated in HBV-LC patients. As a naturally occurring vitamin with the basic structure of menaquinone, vitamin K2 is well-known for its anti-bleeding properties. It also contributes to enhancing hepatic detoxification function, and may play a role in preventing the progression of liver cirrhosis to hepatocellular carcinoma, thereby protecting liver health. This upregulation of the menaquinol biosynthesis in HBV-LC patients might represent a stress response triggered by alterations in coagulation parameters. However, for the HC subjects, their gut microbiota exhibited more active *Bifidobacterium* shunt, pyruvate fermentation, peptidoglycan and glycogen biosynthesis, lactose and galactose degradation, acetate and lactate production, methanogenesis from acetate, these changes were consistent with the changes in the gut microbial composition, saying enriched abundance of SCFA-producing bacteria and *Bifidobacterium* spp. in HC group.

This cross-sectional study focused on HBV-related cirrhosis (HBV-LC) patients in Lanzhou, Gansu Province—a region in Northwest China with a high prevalence of HBV—to investigate the gut microbiota profile and its potential clinical implications. Our research yielded several important and intriguing findings. However, the causal relationship between the altered microbial species and HBV-LC remains to be validated. In future studies, we aim to expand the sample size by including participants from other cities across Northwest China, thereby enhancing the generalizability of the present results to other ethnic or geographic populations. Additionally, we plan to conduct transcriptomic analyses (particularly spatial transcriptomics and whole transcriptome sequencing (Fan et al., 2025; Fan et al., 2024; Liu et al., 2023)), metabolomic profiling, and molecular experiments. These efforts will help validate our current findings, strengthen the reliability of the conclusions, and ultimately facilitate the translation of research outcomes into clinical practice.

## 5 Conclusion

By employing shotgun metagenomic sequencing combined with a metagenome-wide association study, this research identifies significant alterations in both the composition and functional profile of the gut microbiota between patients with HBV-related liver cirrhosis (HBV-LC) and healthy controls in Northwest China. These alterations are characterized by reduced alpha diversity, decreased beneficial bacterial species including *F. prausnitzii*, *B. pseudocatenulatum*, and *B. longum*, enriched opportunistic pathogens such as *E. coli*, *Fusobacterium nucleatum*, *Haemophilus parainfluenzae* and *Veillonella* spp., and microbial metabolic functions reprogramming. This study addresses a current gap in gut microbiome research on HBV-LC patients within the northwest region of China. It provides novel data to support further investigations into the role of gut microbiota in HBV-LC and establishes a theoretical basis for microbiota-targeted interventions aimed at the precise prevention and treatment of HBV-LC.

## Data Availability

The original contributions presented in the study are publicly available. This data can be found here: https://db.cngb.org/data_resources/project/CNP0007660/.

## Supplementary material

SUPPLEMENTARY FIGURE S1 Comparison of the age and BMI between HC and HBV-LC by Student’s t-test and Wilcoxon rank-sum test.

SUPPLEMENTARY FIGURE S2 Comparison of the clinical indexes between HC and HBV-LC by Student’s t-test.

SUPPLEMENTARY FIGURE S3 PCoA based on the Bray–Curtis distances at species level.

SUPPLEMENTARY FIGURE S4 PCoA analysis based on the Bray–Curtis distances at functional pathway level.

SUPPLEMENTARY FIGURE S5 Spearman’s rank correlation between significantly different species and clinical indicators in all the 86 samples.

SUPPLEMENTARY TABLE S1 The basic information of the two groups of people.

SUPPLEMENTARY TABLE S2 The detailed clinical data of the subjects.

SUPPLEMENTARY TABLE S3 The relative abundance of all gut microbial taxon.

SUPPLEMENTARY TABLE S4 PERMANOVA of phenotypes on gut microbiota.

SUPPLEMENTARY TABLE S5 The Shannon, Simpson, and inverse-Simpson indexes of all samples.

SUPPLEMENTARY TABLE S6 The relative abundance of the gut microbial functional pathways.

SUPPLEMENTARY TABLE S7 Spearman’s rank correlation analysis between significantly different species and clinical indexes.

SUPPLEMENTARY TABLE S8 Significantly different genera, species, and functional pathways (P < 0.05) that were not displayed in the Figure 2 and Figure 3.

SUPPLEMENTARY TABLE S9 Raw sequencing data experienced filtering and removal of host reads to obtain high-quality clean reads for taxonomic and functional annotation.
