# Supplementary figures and images for: A metagenome-wide association study of gut microbiota in hepatitis B virus-related cirrhosis in northwest China
Source: Front Genet. 2025 Aug 20;16:1619911. doi: 10.3389/fgene.2025.1619911 (PMC12404923; doi:10.3389/fgene.2025.1619911)

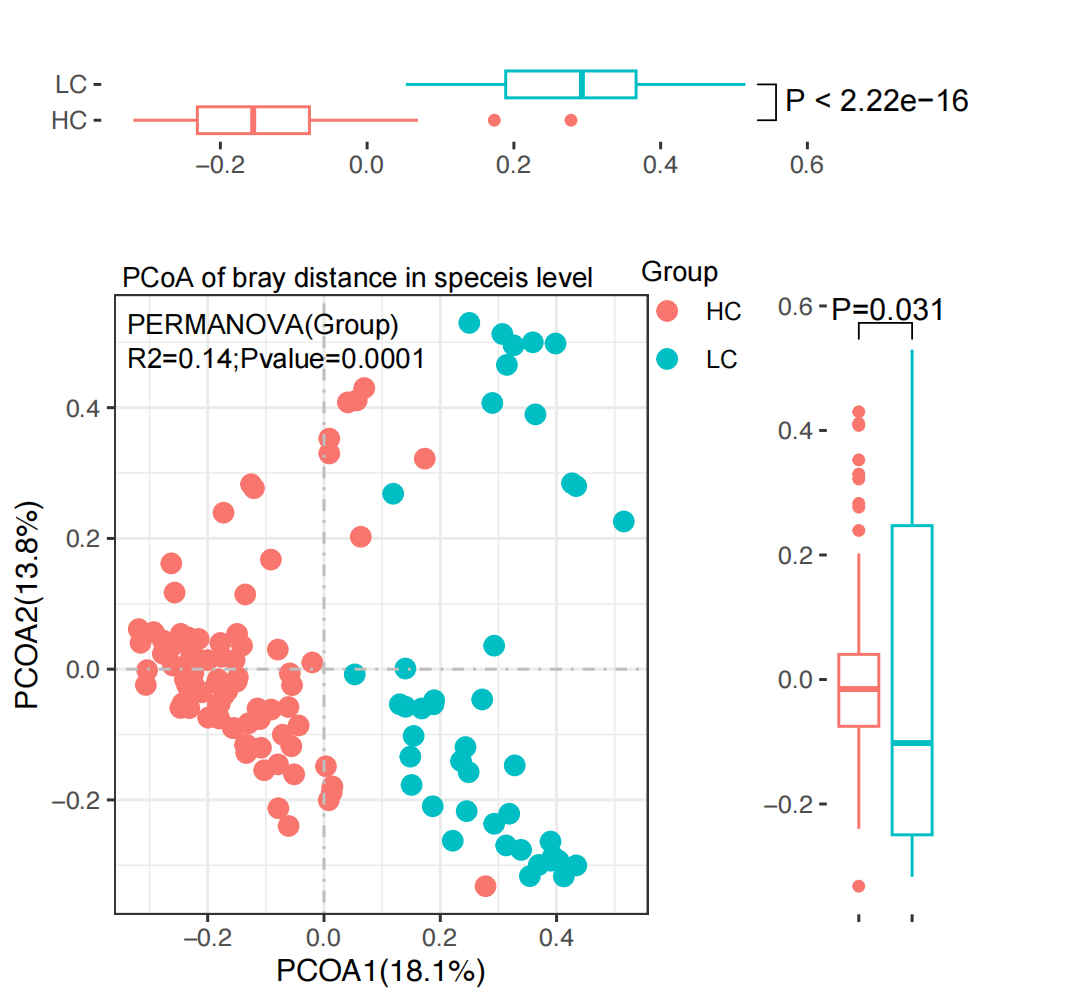

Supplement: Supplementary file 1 [file Image3.tif]

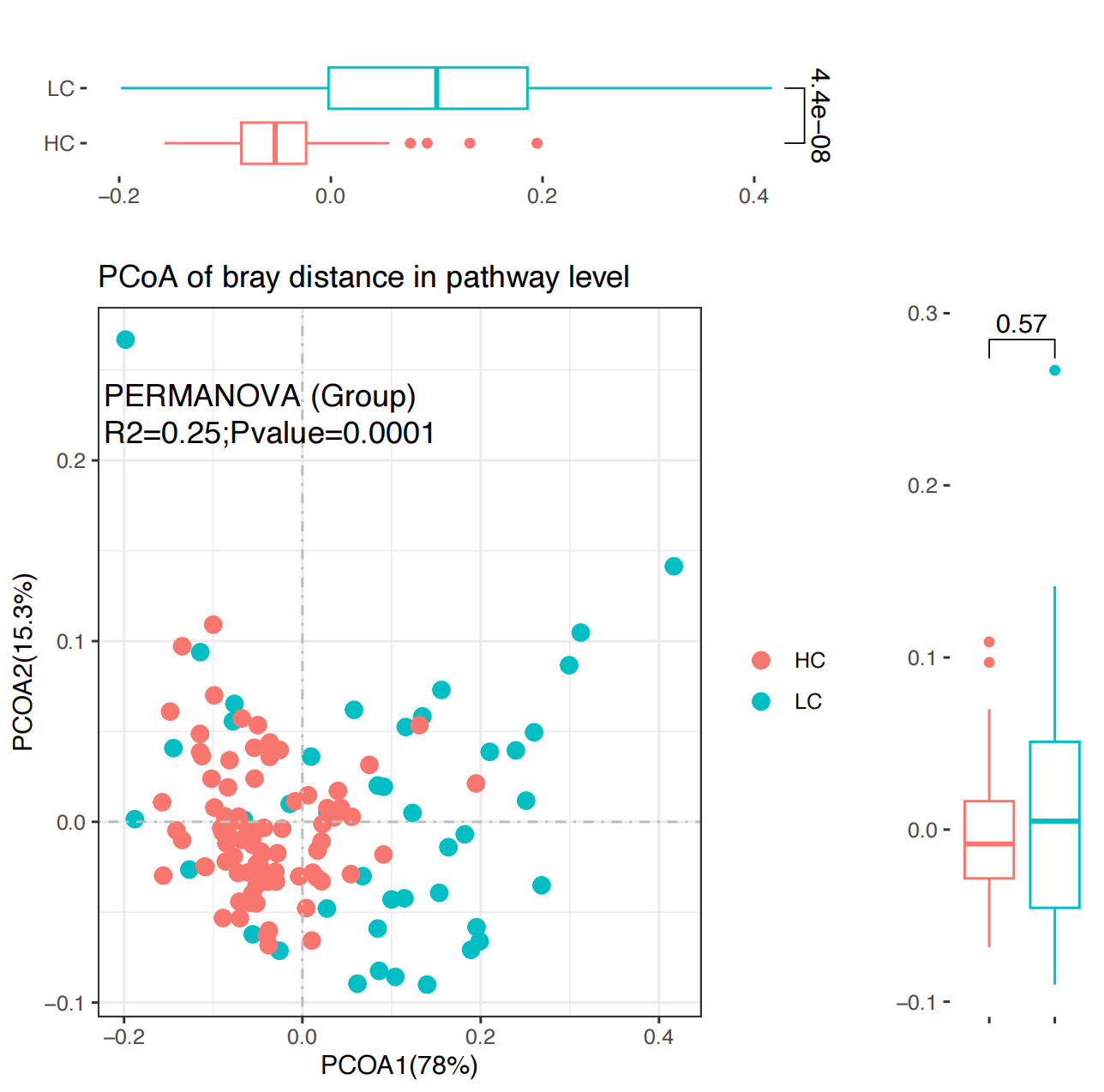

Supplement: Supplementary file 2 [file Image4.tif]

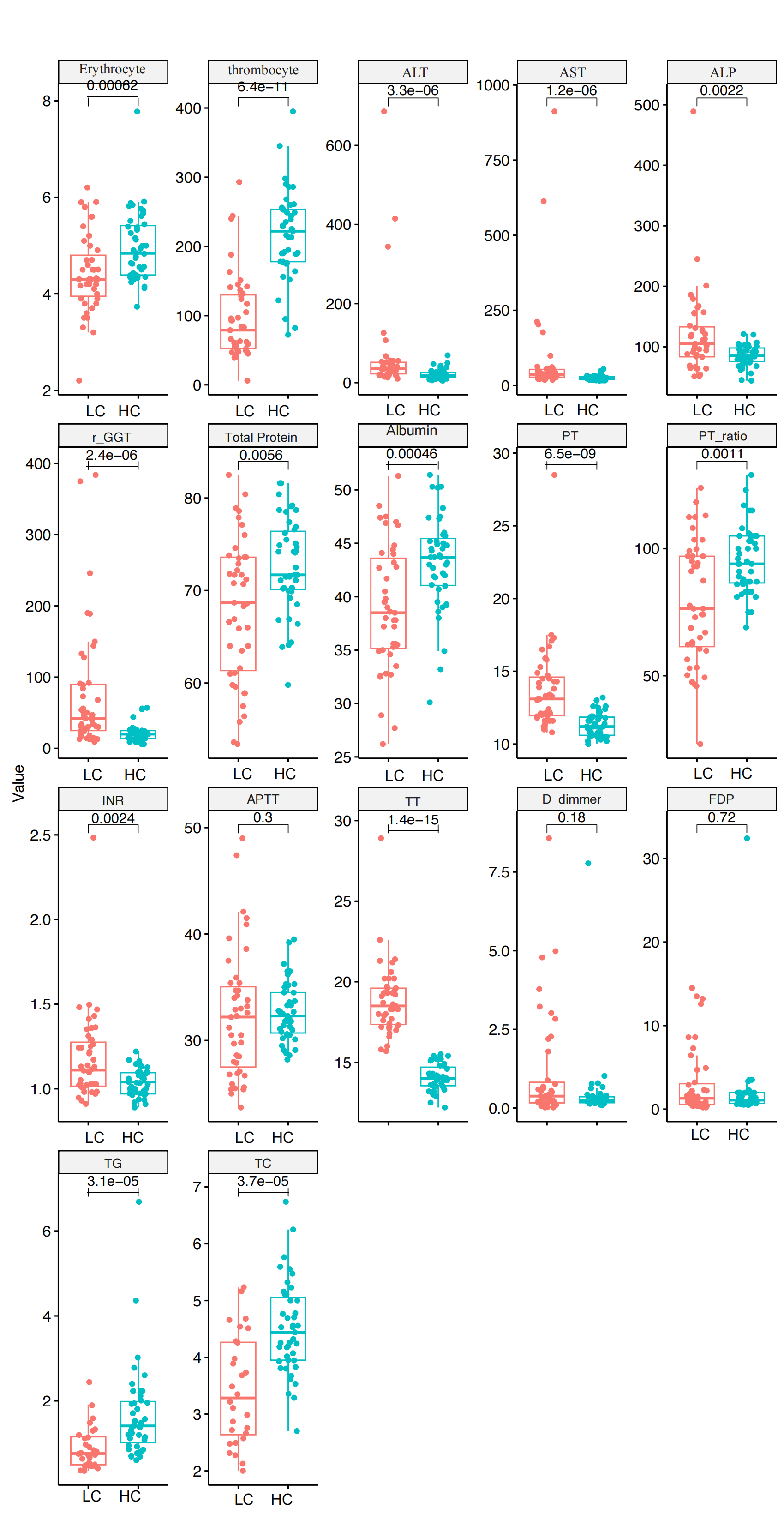

Supplement: Supplementary file 3 [file Image2.tif]

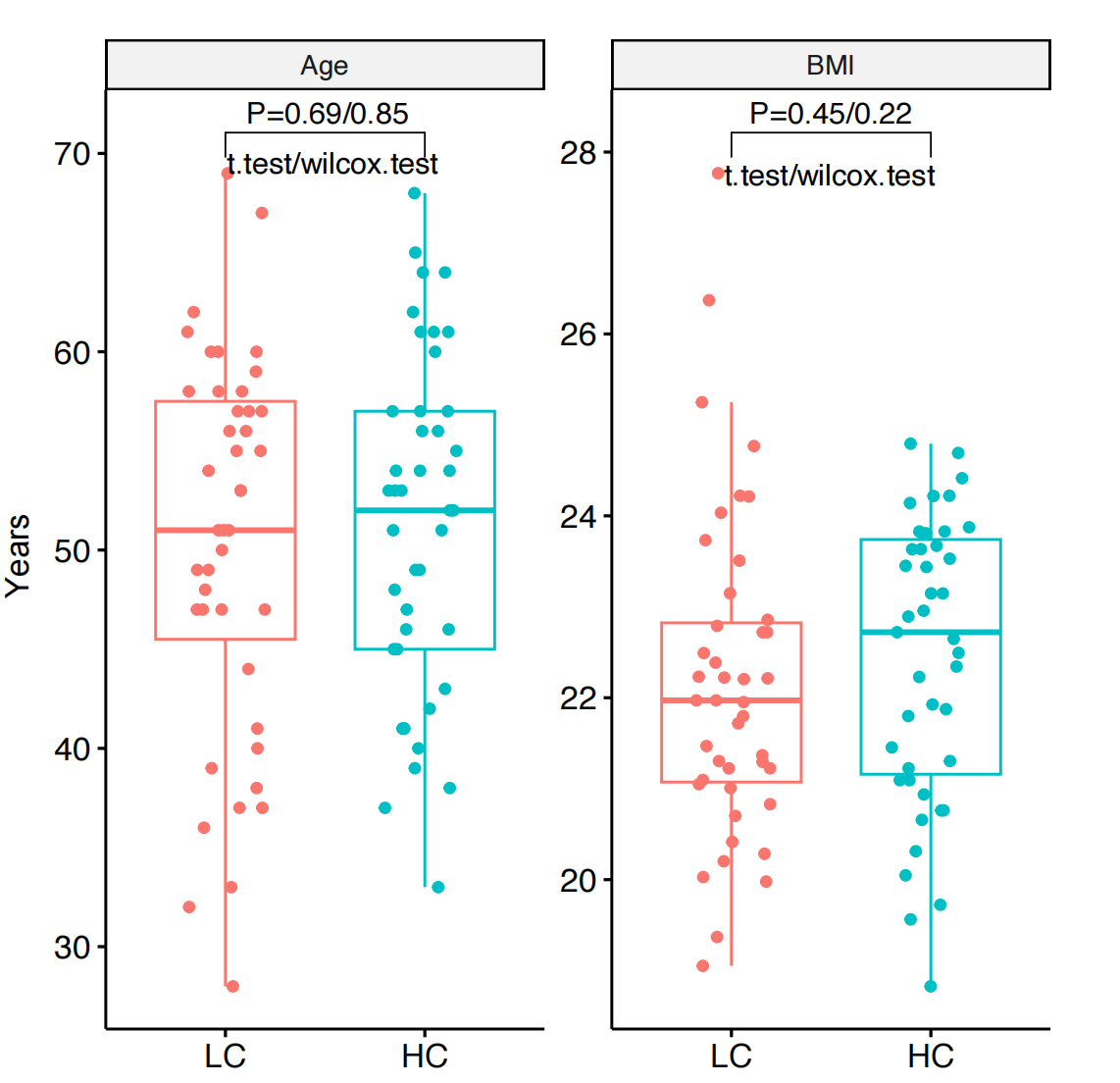

Supplement: Supplementary file 4 [file Image1.tif]

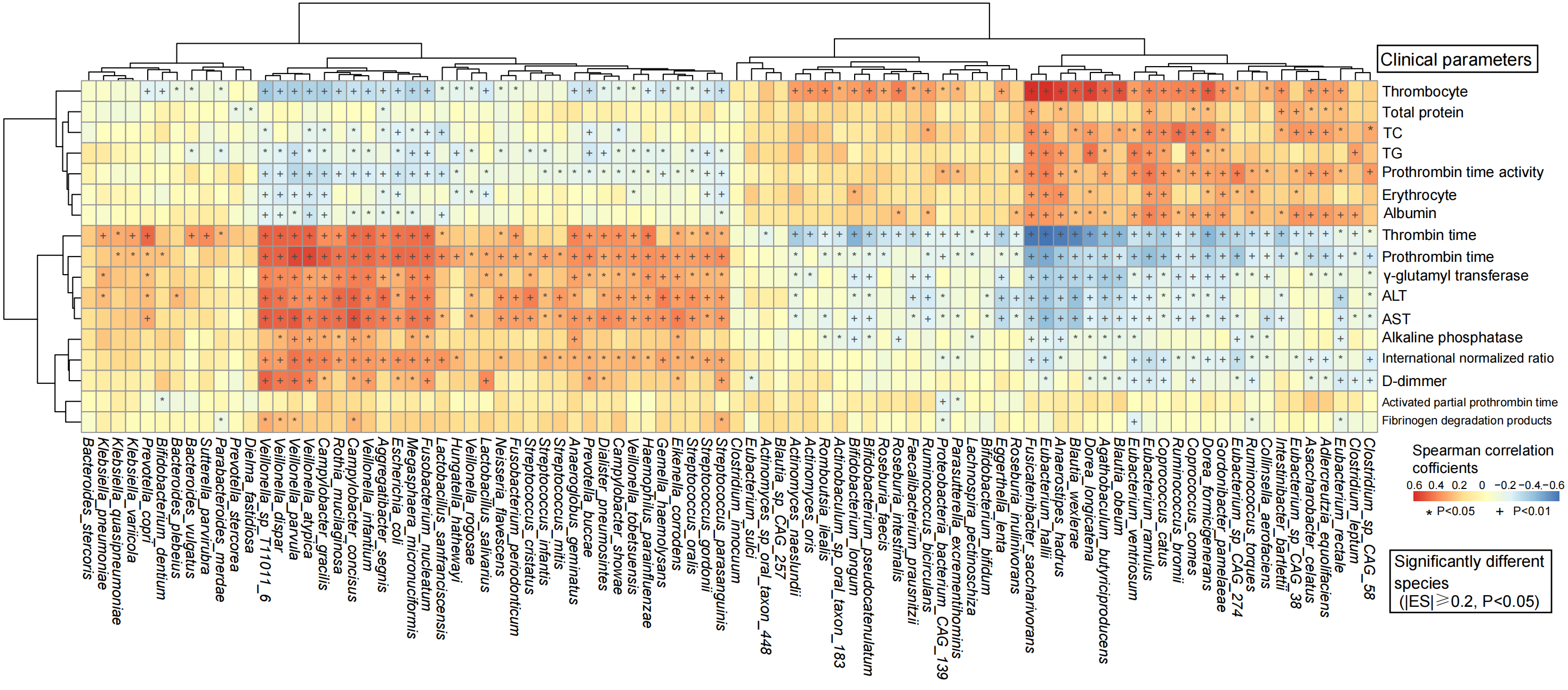

Supplement: Supplementary file 6 [file Image5.tif]
